# Novel encoding and updating of positional, or directional, spatial cues are processed by distinct hippocampal subfields: Evidence for parallel information processing and the “what” stream

**DOI:** 10.1002/hipo.22833

**Published:** 2018-02-19

**Authors:** Thu‐Huong Hoang, Verena Aliane, Denise Manahan‐Vaughan

**Affiliations:** ^1^ Medical Faculty, Department of Neurophysiology Ruhr University Bochum Bochum 44780 Germany; ^2^ International Graduate School of Neuroscience Ruhr University Bochum Bochum 44780 Germany

**Keywords:** fluorescence *in situ* hybridization, hippocampus, immediate early gene, information encoding, spatial learning

## Abstract

The specific roles of hippocampal subfields in spatial information processing and encoding are, as yet, unclear. The parallel map theory postulates that whereas the CA1 processes discrete environmental features (positional cues used to generate a “sketch map”), the dentate gyrus (DG) processes large navigation‐relevant landmarks (directional cues used to generate a “bearing map”). Additionally, the two‐streams hypothesis suggests that hippocampal subfields engage in differentiated processing of information from the “where” and the “what” streams. We investigated these hypotheses by analyzing the effect of exploration of discrete “positional” features and large “directional” spatial landmarks on hippocampal neuronal activity in rats. As an indicator of neuronal activity we measured the mRNA induction of the immediate early genes (IEGs), Arc and Homer1a. We observed an increase of this IEG mRNA in CA1 neurons of the distal neuronal compartment and in proximal CA3, after novel spatial exploration of discrete *positional* cues, whereas novel exploration of *directional* cues led to increases in IEG mRNA in the lower blade of the DG and in proximal CA3. Strikingly, the CA1 did not respond to directional cues and the DG did not respond to positional cues. Our data provide evidence for both the parallel map theory and the two‐streams hypothesis and suggest a precise compartmentalization of the encoding and processing of “what” and “where” information occurs within the hippocampal subfields.

## 1 INTRODUCTION

The hippocampal formation plays an important role in spatial learning triggered by the exploration of a new environmental context and its contents. According to the parallel map theory (Jacobs, 2003; Jacobs & Schenk, 2003), the CA1 generates a “sketch map” in which positional cues are encoded. In contrast, the dentate gyrus encodes a “bearing map” in which directional cues are encoded. Finally, these subsets of spatial information are integrated in the CA3 region to form a complete map (Jacobs, 2003; Jacobs & Schenk, 2003). The two‐streams hypothesis postulates that information related to an item's feature and spatial characteristics are encoded in two separate streams in the parahippocampal region: the “what” stream and “where” stream, with this information being then integrated in the hippocampus (Mishkin, Ungerleider, & Macko, 1983). Anatomical and functional data suggest that such a distinction between the “what” and “where” pathway can also be observed in the CA1 and CA3 regions (Amaral & Witter, 1989; Burke et al. 2011; Henriksen et al. 2010; Ishizuka, Weber, & Amaral, 1990; Ito & Schuman, 2012; Sauvage, Nakamura, & Beer, 2013; Witter, 2007; Witter, Wouterlood, Naber, & van Haeften, 2000). The distal CA1 and proximal CA3 regions may process information from the “what” stream and the proximal CA1 and distal CA3 region may process information from the “where” stream. Furthermore, a differentiation of the “what” and “where” streams within the dentate gyrus may also occur (Chawla et al. 2005).

Hippocampal synaptic plasticity is likely to comprise the cellular process through which the hippocampus enables spatial learning (Kemp & Manahan‐Vaughan, 2008), by expressing two forms of persistent synaptic plasticity, long‐term potentiation (LTP) and long‐term depression (LTD) (Kemp & Manahan‐Vaughan, 2004; Manahan‐Vaughan & Braunewell, 1999). Whereas changes in global space facilitate the expression of persistent LTP (Hagena & Manahan‐Vaughan, 2012; Kemp & Manahan‐Vaughan, 2004), novel experience of environmental content facilitates LTD (Kemp & Manahan‐Vaughan, 2004, 2007, 2008). Strikingly, novel exposure to discrete visuospatial, audiospatial or olfactospatial cues facilitates LTD in the CA1 region (Dietz & Manahan/Vaughan, 2017; André & Manahan‐Vaughan, 2013; Kemp & Manahan‐Vaughan, 2004), whereas novel exposure to large landmark cues facilitates LTD in the dentate gyrus (Kemp & Manahan‐Vaughan, 2008). These findings are in line with both the parallel map theory and two streams hypothesis.

In this study, we implemented behavioral learning paradigms that facilitate the expression of robust hippocampal LTD to explore the functional basis of both hypotheses. We used fluorescence *in situ* hybridization (FISH), to map the activity‐dependent mRNA expression of the immediate early gene Arc in the hippocampus, which is related to learning and synaptic plasticity (Bramham, Worley, Moore, & Guzowski, 2008; Guzowski, McNaughton, Barnes, & Worley, 1999; Guzowski et al. 2005; Korb & Finkbeiner, 2011; Link et al. 1995; Lyford et al. 1995; Vazdarjanova, McNaughton, Barnes, Worley, & Guzowski, 2002). This technique allows the analysis of different hippocampal subregions in the same animal. We observed learning‐induced increases of Arc mRNA expression in the CA1 and CA3 regions after exploration of small partially concealed environmental features, whereas an increase in the dentate gyrus and CA3 region occurred after exploration of large highly visible spatial landmarks. The former effects were most prominent in the distal CA1 and proximal CA3, whereas the latter effects were significant in the lower blade of the dentate gyrus and also in the proximal CA3 region. These findings empirically support both the parallel map and two‐streams theories of hippocampal function.

## 2 MATERIALS AND METHODS

The study was conducted in accordance with the European Communities Council Directive of September 22nd, 2010 (2010/63/EU) for care of laboratory animals and after approval of the local ethics committee (Landesamt für Natur‐, Umwelt und Verbraucherschutz, Nordrhein Westfalen). All efforts were made to minimize the number of rats used for this study. The animals were housed in groups in a temperature and humidity controlled vivarium with constant 12‐hr light‐dark cycle (lights on from 7 a.m. to 7 p.m.). The animals had *ad libitum* access to food and water.

### 2.1 Behavioral experiments

**Figure 1:**
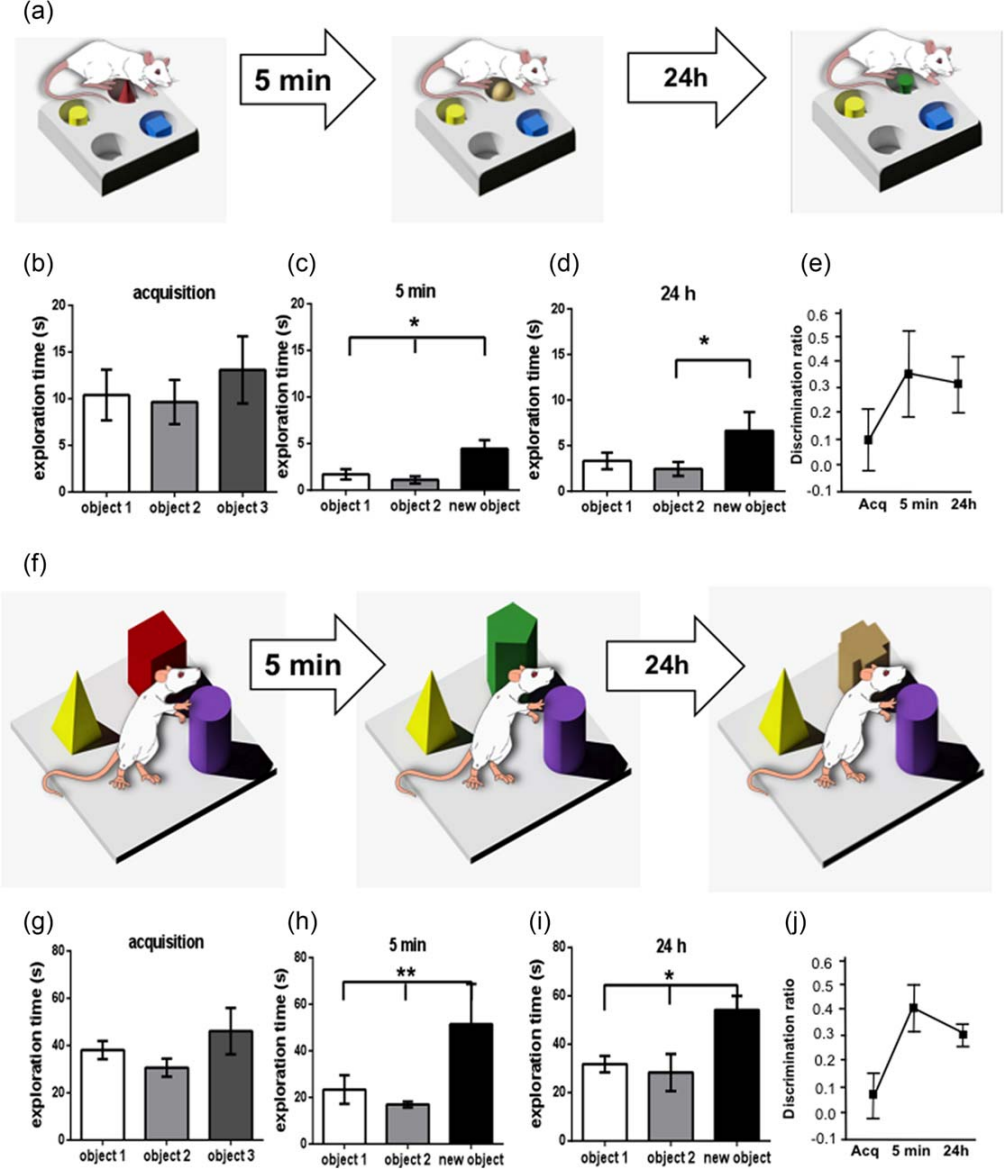
Spatial object recognition is evident 5 min and 24 h after acquisition. Animals were exposed to three small novel objects (positional cues) that were placed within three of four holeboard holes (a), or to three large novel objects (directional cues) that were placed on the floor of the recording chamber (f). To assess if the animals created a memory for these objects we tested for object recognition 5 min (test phase 1) and 24 h (test phase 2) after the initial novel object exposure (acquisition phase). Here, two, now familiar, objects were retained and one object was always novel. Object exploration time was assessed as a percentage of the total exploration time. In the positional cues group no statistically significant difference in object preference was observed during acquisition (b). Significantly higher exploration recognition of the new object both 5 min (c) and 24 h (d) after acquisition (Acq), and object discrimination ratios (e) confirmed that the animals had formed memories of the “familiar” objects. This was also the case in the landmark cue group (F–J). Here, no preference was evident between objects 1, 2, and 3 during the acquisition phase (g). Importantly, recognition of the new object occurred 5 min (h) and 24 h (i) after acquisition (Acq), as reflected by a significantly higher exploration of the new object compared to either object 1 or object 2, and by and landmark discrimination ratios (j). Bonferroni or Fisher LSD posthoc test: *p < .05 and **p < .01 [Color figure can be viewed at http://wileyonlinelibrary.com]

In a gray washable Perspex chamber that measured 40 × 40 × 40 cm (Kemp & Manahan‐Vaughan, 2004), male adult Wistar rats (7–9 weeks old, *n* = 35) underwent an acquisition phase for 5 min, a first recognition test after 5 min (short term memory) and a second recognition test after 24 h (long term memory). Two different tests were performed (Figure 1a,f). One test comprised learning about discrete positional cues that involved exploration of small objects that were placed in holeboard holes and required that the animals be physically on top of the holes in order for the items to be detected and perceived (Figure 1a). This task facilitates LTD in the hippocampal CA1 region, but not in the dentate gyrus (Kemp & Manahan‐Vaughan, 2008; Manahan‐Vaughan & Braunewell, 1999). The other test comprised learning about large landmarks placed in the test arena, that served as directional cues for navigation, and that could be viewed from afar (Figure 1f). This task facilitates LTD in the dentate gyrus, but not in the CA1 region (Kemp & Manahan‐Vaughan, 2008). Changing the object configurations, or replacing a familiar object with a novel object also leads to LTD and these effects are tightly linked to learning about the new spatial configuration and context (Goh & Manahan‐Vaughan, 2013; Kemp & Manahan‐Vaughan, 2008).

Before all behavioral experiments, the animals were handled once for approximately 15 min per day for several days. The animals were habituated to the test chamber for 1 h per day, for two consecutive days immediately prior to commencement of the study. On the day of the first experiment, the animals were placed in the chamber for 1 h before the acquisition phase was commenced. During the acquisition phase, one cohort of animals was presented with three small novel objects (4 × 2 × 2 cm) that were placed (one each) inside three of the four holeboard holes. The objects did not extend above the surface of the holeboard and the rats had to put their noses inside the holes to detect them. In a second cohort the animals were exposed to three large “landmark” objects (11 × 10 × 10 cm) that were placed on the surface of the floor of the chamber and could be seen from afar.

After 5 min of exploration (starting from the beginning of the first explorative activity at one of the objects), the objects were removed. A further 5 min later the animals were exposed to two of the familiar objects and one novel object, without changing the original spatial locations of the objects (Test phase 1). The following day, 24 h later, the animals were reintroduced to the chamber and after acclimatizing for 1 h, they were shown the same two familiar objects from the day before along with one *completely* novel object (Test phase 2).

The object that was removed after the acquisition phase was pseudorandomized across the rats to control for a positional bias. However, in the results section the familiar objects are always referred to as object 1 and object 2. During the acquisition phase and test phases 1 and 2, the behavior of the animals was videotaped and the time spent exploring each object was recorded. In the case of the landmarks we defined exploring an object as pointing the nose to the object at a distance of <1 cm and/or touching it with the nose. For the positional cue (holeboard objects) group, exploration time was assessed as being the time during which the rats actively pointed their noses inside the hole in which the object was situated.

### 2.2 *In situ* hybridization

Before the experiments, the animals were handled during two consecutive days, for approximately 15 min per day, by the experimenter. Following this procedure, animals were habituated to the chamber (40 × 40 × 40 cm) in which the behavioral experiments were performed for two consecutive days. On each day of habituation, the animals were placed into the chamber for 60 min and then returned to their home cage.

Where effects of the positional cues on IEG mRNA expression were tested, brains were removed 5–6 min after exposure of the animals to the objects in the holeboard and Arc neuronal mRNA changes were analyzed (see below).

In the landmark (directional cue) study, we assessed both Arc and Homer1a expression, given the fact that the neuronal Arc mRNA expression was found (in our study) to be relatively low in the DG. Homer1a expression was thus used as a means to verify the DG results obtained using Arc. In one animal cohort, brains were removed 5–6 min after exposure to the novel landmarks, so that could examine exploration‐dependent Arc mRNA expression. In a second cohort, brains were removed ca. 40 min after exposure to the novel landmarks. Activity‐dependent elevations of Arc mRNA exhibit peak levels 5–6 minutes after the experience (Guzowski et al., 1999), whereas somatic Homer1a peaks 35–40 min after the experience (Vazdarjanova et al. 2002).

On the day of the experiment, the animals were placed into the chamber 60 min before the behavioral experiment started. The following groups were differentiated:

1. Control group: no learning event, brain removal after the animal resided in the chamber for 60 min;
2. positional cue group: brain removal 5–6 min after commencement of novel exploration (Arc mRNA assessment);
3. landmarks group: brain removal either 5–6 min (Arc mRNA assessment) or ca. 40 min after commencing novel exploration (Homer1a mRNA assessment).

A different object configuration was randomly chosen for each animal. During exploration, the behavior of the animals was videotaped and the exploration time for each quadrant was subsequently analyzed. The same number of animals of the groups to be compared (control vs. positional cues and control vs. landmarks) were put in the chambers at the same time and the behavioral experiments were performed on the same day.

For *in situ* hybridization, brains were removed, shock‐frozen for 2 min in isopentane at −20 °C and stored at −80 °C until further processing. Later, 20 µm thick coronal sections (three slices per glass slide) containing the hippocampus (from ∼3.6 to 4 mm from Bregma) were cut on a Cryostat (Leica CM 3050S), mounted directly on superfrost plus slides (Gerhard Menzel GmbH, Braunschweig, Germany) and stored at −80 °C until further processing.

Fluorescin‐labeled probes were created using the Ambion MaxiScript Kit (Invitrogen, Carlsberg, USA). The Arc cDNA plasmid was prepared by Entelechon (Bad Abbach, Germany) using a ∼3 kb Arc transcript according to the sequence of Lyford et al. (1995). The Homer1a cDNA plasmid was prepared by Entelechon (Bad Abbach, Germany) using a ∼1.2 kb Homer1a transcript according to the sequence of Brakemann et al. (1997). The cRNA probes were prepared from the linearized cDNA using the Ambion MaxiScript Kit and a premixed RNA labeling nucleotide mix containing the Digoxigenein‐ and Biotin‐ labeled UTP (Invitrogen, Carlsberg, USA). After purification on Mini Quick Spin RNA columns (Roche Diagnostics, Mannheim, Germany) we verified the yield and integritiy of the RNA probes using gel electrophoresis.

At the time‐point of further processing, one glass slide per animal (with three slices each) was chosen and left at room temperature until the slices were defrosted. From each animal the glass slide with slides from the dorsal hippocampus at ∼3.8 mm from Bregma was chosen. We then applied the following protocol for the Arc and Homer1a mRNA *in situ* hybridization (adapted from Guzowski et al., 1999): The slices were fixed in ice cold 4% paraformaldehyde in PBS (fresh and filtered) for 10 min and then washed in 2× saline‐sodium citrate buffer (SSC) for 2 min. The slices were left in acetic anhydride solution for 10 min, quickly washed four times each 1 min in 2× SSC and left in 2× SSC finally for 5 min. The humid chamber was prepared with 2× SSC/50 deionized Formamid (Sigma‐Aldrich, 47671‐250ML‐F) (à/à) soaked filter paper. 100 µL of 1× prehybridization buffer (Sigma‐Aldrich, P1415‐50ML) was applied on each slide for 30 min at room temperature (RT). The slides were covered with a piece of Parafilm^®^ to prevent from drying out. The fluorescein‐labeled DNA probe was diluted with a concentration of 1 ng/1 µL in 1× hybridization buffer (Sigma‐Aldrich, H7140‐50ML), heated at 90 °C for 5 min and applied on the brain sections. The slices were again covered with Parafilm^®^ and hybridized for approximately 17 h in a humid chamber at 56 °C. Following the hybridization, stringent washing steps were conducted using SSC buffer in different concentrations that had been prepared in different concentrations and stored either at 37 or 56 °C a day before to reach the optimal temperature. The Parafilm^®^ was removed and the slides were placed in 2× SSC at 56 °C three times, each for 5 min. RNase (Sigma‐Aldrich, R6513‐10MG) was solved in 2× SSC at a concentration of 100 µg/100 mL 2× SSC and stored at 37 °C. The slides were placed into the RNase‐solution for 15 min at 37 °C, followed by 10 min 2× SSC buffer at 37 °C. The stringent washing was continued in 0.5× SSC for 10 min at 56 °C, in 0.5× SSC for 30 min at 56 °C, in 0.5× SSC for 10 min at room temperature (RT), 1× SSC for 2× 5 min at RT and finally rinsed in TBS three times, each for 5 min at RT.

The signal detection was carried out by means of immunohistochemistry. The Homer1a mRNA signal was detected by Streptavidin Cy2 (Dianova, Cat#016220084) [1:250] in 1% BSA (Sigma‐Aldrich, Cat#A3912) in TBS‐Tween (Polysorbate 20) for 30 min after a blocking step in 1% BSA in TBS‐Tween for 70 min. After three washes of five minutes duration in TBS, the signal was amplified using biotinylated Anti‐Streptavidin (Vector Labs, Cat# BA0500) [1:100] in 1% BSA in TBS‐Tween. The slices were incubated for 20 min and then washed three times each for 5 min with TBS. The Homer1a signal was visualized using StreptAvidin Cy2 [1:250] in 1% BSA in TBS‐Tween 20, incubating the slices for 90 min. The Arc mRNA signal detection steps were conducted as following: the slices were pretreated with 3% H_2_O_2_ in 1× SSC for 15 min, washed 3× 5min in TBS, then incubated for 70 min with the blocking solution 1% BSA in TBS‐Tween 20 + 20% Avidin (Vector Labs, Cat# SP2001). Arc‐Digoxigenin was detected by Anti‐Digoxigenin‐POD Fab fragment from sheep (Roche, Cat #11207733910, RRID:AB_514500) 1:400 in 1% BSA in TBS‐Tween + 20% Biotin (Vector Labs, SP2001) for 90 min. The signal was enhanced by using biotinylated Tyramine (Adams, 1992) in TBS for 20 min and the slices were then washed four times each 2 min in TBS. The Arc mRNA signal was visualized using Streptavidin Cy5 [1:2,000] (Dianova, Cat#016170084) and the nuclei was visualized by using 4′,6‐diamidino‐2‐phenylindole [1:10,000] (DAPI, Invitrogen) in 1% BSA in TBS‐Tween 20 for 90 min at RT. The slices were washed again three times each 5 min in TBS, and quickly washed with distilled water, air dried overnight and mounted in Dianova Mounting medium (Dianova SCR‐38447).

### 2.3 Data analysis

To analyze the behavioral data, we determined the exploration time per object as described above. The exploration time is given in seconds (s) ± standard error of the mean (s.e.m.). We also calculated the discrimination ratio to compare familiar versus novel object exploration. Here, the exploration time of the familiar object, or the novel object was divided by the total exploration time (both objects). A positive ratio indicates that more time was spent exploring the novel object and thus, that the animal realizes that the object hasn't been seen before.

For statistical analysis (using Statistica, RRID:SCR_014213) we performed a repeated measures analysis of variance (ANOVA) with OBJECT (object 1 vs object 2 vs object 3, or object 1 vs. object 2 vs. new object) as a repeated measures factor and conducted post‐hoc analysis using the Bonferroni, or Fisher LSD, test. To compare the exploration time during the acquisition phase in the behavioral study with the exploration time during the acquisition phase in the *in situ* hybridization experiments we performed a multivariate ANOVA with the factor OBJECT (object 1 vs. object 2 vs. object 3) as a repeated measures factor and the factor EXPERIMENT (behavior vs. *in situ* hybridization) as the between groups factor.

**Figure 2:**
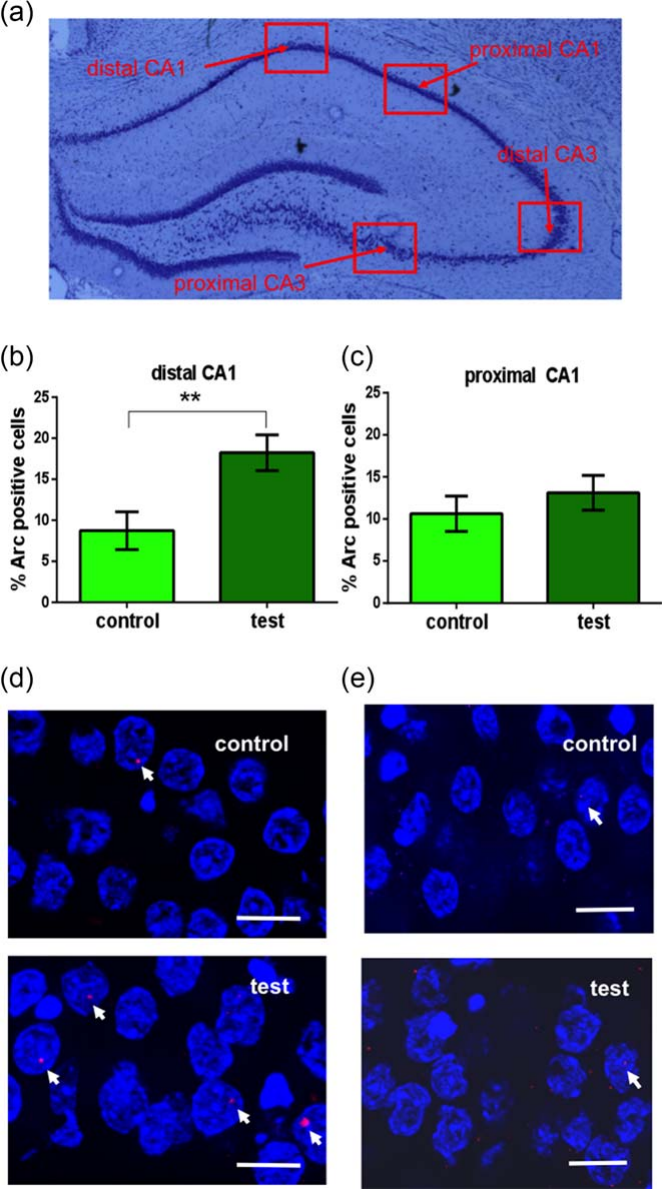
Arc mRNA expression is specifically increased in the distal CA1 after the exploration of discrete spatially arranged objects. (a) Nissl stained sections of the rat hippocampus showing (red squares) the regions analyzed in CA1 and CA3. The exploration of novel objects that were placed inside, and below, the surface of 3 of 4 holeboard holes (positional cues) results in increased Arc mRNA expression in the distal CA1 (b) but not in the proximal CA1 (c). One way ANOVA: **p < .01. (d, e) Photomicrographs show Arc mRNA expression (red points, indicated by arrows) in the distal CA1 and proximal CA1 regions of control animals (control) or animals that participated in positional cue exploration (test). Blue: nuclear staining with DAPI. Images were taken using a 63× objective. Scale bar: 10 μm [Color figure can be viewed at http://wileyonlinelibrary.com]

In order to analyze Arc or Homer1a mRNA labeling within the nuclei of the pyramidal cells of the CA1, CA3 and the granule cells of the dentate gyrus, we obtained Z‐stacks at a 63× magnification using a Zeiss ApoTome. For each animal, three consecutive slices were used for *in situ* hybridization, whereby we analyzed both hemisphere of each slice and calculated the mean of these three slices. Furthermore, for each slice we analyzed the proximal and distal subcompartments of the CA1 and CA3 regions and the upper and lower blades of the dentate gyrus by obtaining Z‐stacks of these area (see Figure 2a). The proximal and distal areas of the CA1 and CA3 regions were selected as described by others (Nakamura, Flasbeck, Maingret, Kitsukawa, & Sauvage, 2013). Using “Fiji” software (Schindelin et al., 2012, Fiji, RRID:SCR_002285) we first marked all complete nuclei that were not cut on the edges either in the *x*, *y*, or *z* direction. Afterwards, each nucleus was checked for Arc or Homer1a mRNA and the percentage of Arc or Homer1a mRNA positive cells was calculated. The examiner who analyzed the data was blind to the different animal groups. We assessed effects of novel experience of discrete close‐range environmental cues and of novel exposure to landmark navigation‐relevant cues.

For statistical analysis we used the Statistica software and performed one‐way ANOVA and multifactorial ANOVA with the factor “cues” as the between groups factor to analyze the effect of exposure to novel cues on exploration behavior across hippocampal subregions. A Bonferroni post‐hoc test was used to assess subregion‐specific effects between control and test‐animals.

## 3 RESULTS

### 3.1 Memory of positional and directional cue learning is retained for at least 24 hr

This study aimed to clarify where information, in the form of discrete positional, or landmark (directional) cue information is processed in the hippocampus. For this, we first needed to confirm that our behavioral tests generated memories in the rodents that lasted for at least 24 h, in line with the creation of robust (and not transient) memories.

To analyze to what extent the rats explored and remembered the positional cues (Figure 1a–e), we exposed assessed the percentage of the total exploration time spent exploring all three novel cues during the acquisition phase, test phase 1 (one cue exchanged for a novel cue) that occurred 5 min after the acquisition phase and test phase 2 (one cue exchanged for a novel cue) that occurred 24 h after the acquisition phase. During the acquisition phase (novel exposure to three unfamiliar objects), the exploration times were similar for animals exposed to positional cues (*n* = 10) (repeated measures ANOVA: *F*_2,18_ = 0.4113, *p* > .05; Figure 1b). Exposure to two familiar and one novel object in test phase 1 (5 min after acquisition, Figure 1c), showed that the animals exhibited an exploration preference for the new object (repeated measures ANOVA: *F*_2,18_ = 9.95929, *p* < .01 Bonferroni: object 1 vs. new object: *p* < .01; object 2 vs. new object: *p* < .01). When the animals re‐experienced the familiar objects in conjunction with another entirely unfamiliar objects 24 h after the acquisition trial (test phase 2, Figure 1d), they also exhibited a significant preference of the novel object (repeated measures ANOVA: *F*_2,18_ = 4.66089, *p* < .05; Bonferroni: object 1 vs. new object: *p* < .05; object 2 vs. new object: *p* < .05). Effects were confirmed by calculation of object discrimination ratios (Figure 1e, one‐way ANOVA, *p* < .05). These results confirm that the animals remembered the positional cues for at least 24 h.

We then ran the same kind of assessment of the landmark cues (Figure 1f–j). During the acquisition phase, no preference between object 1, object 2 and object 3 was evident (*n* = 8, Figure 1g) (Fisher LSD, *p* > .05). When the animals explored the novel landmark 5 min after acquisition (test phase 1), a significant preference of the new object over objects 1 and 2 was apparent (Figure 1h) (Fisher LSD: object 1 vs. new object: *p* < .01; object 2 vs. new object: *p* < .01). Similarly, 24 h after the acquisition phase (test phase 2) significantly higher exploration of the novel object occurred (Figure 1i; Fisher LSD: object 1 vs. new object: *p* < .05; object 2 vs. new object: *p* < .05). Effects were confirmed by calculation of object discrimination ratios (Figure 1j, one‐way ANOVA, *p* < .05). Thus, the animals created a memory of the landmark cues that lasted for at least 24 h.

### 3.2 Exposure to novel positional cues lead to elevations of neuronal immediate early gene mRNA in distal but not proximal neurons of the CA1 region, and in the proximal but not distal CA3 region. The dentate gyrus does not respond to positional cues

The distal CA1 and proximal CA3 are believed to comprise part of the “what” stream, whereas the proximal CA1 and distal CA3 are believed to be part of the “where” stream (Sauvage et al., 2013; Witter, 2007; Witter et al., 2000). To what extent the dentate gyrus can discriminate information of this kind is unclear (Chawla et al, 2005; Tamamaki, 1997; Wyss, 1981). To scrutinize this in detail, we explored to what extent our positional (“what”) and landmark cues (that contain a combination of “what” and “where” information) elicited differentiated expression responses in these hippocampal subfields.

First, we assessed the effect of novel exposure to positional cues on neuronal Arc mRNA levels in the CA1 region (*n* = 8), CA3 region (*n* = 8), and dentate gyrus (*n* = 8). In general, when the percentage of Arc mRNA positive cells was compared between the CA1 (average number of cells analyzed: 18.79 ± 0.44) and the DG (average number of cells analyzed: 76.61 ± 1.85), a significantly greater Arc effect was detected in the CA1 region (Bonferroni test: *p* < 0.05) compared to expression levels in the dentate gyrus (not shown). Furthermore, we also observed a significant elevation of Arc mRNA expression following exploration of novel cues in the CA3 region (average number of cells analyzed 11.45 ± 0.38; Bonferroni test: *p* < .05) compared to the Arc mRNA expression in the dentate gyrus.

In the CA1 region (*n* = 8), we observed a significant overall increase in Arc mRNA expression following novel positional cue exposure (one way ANOVA: *p* < .05 vs. controls, *n* = 8, not shown). But when we subdivided the CA1 region into distal and proximal compartments, our analysis revealed that the significant elevations in neuronal Arc mRNA derived from a *specific* response of neurons in the distal CA1 region (Figure 2b,d) (average number of cells analyzed: 18.22 ± 0.66) (one way ANOVA: *F*_1,14_ = 8.97596, *p* < .01) compared to control animals. Proximal neurons did not respond to the novel positional cues (average number of cells analyzed: distal: 19.36 ± 0.57) (one way ANOVA: *p* > .05 vs. controls, Figure 2c,e).

**Figure 3:**
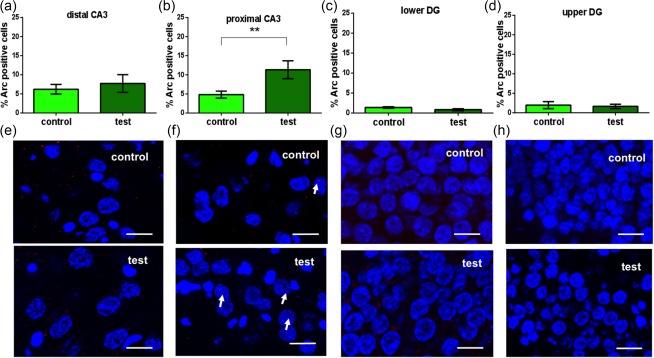
Exploration of positional cues increases Arc mRNA expression in the proximal CA3 region. The distal CA3 region and dentate gyrus are unaffected. Arc mRNA expression was significantly in the proximal CA3 region following exposure to positional cues (b) but not in the distal CA3 region (a) or lower (c) and upper (d) blades of the dentate gyrus. One way ANOVA: **p < .01. (e–h) Photomicrographs show Arc mRNA expression (red points, indicated by arrows) in the CA3 regions and the dentate gyrus of control animals (control) or animals that participated in positional cue exploration (test). Blue: nuclear staining with DAPI. Images were taken using a 63× objective. Scale bar: 10 μm [Color figure can be viewed at http://wileyonlinelibrary.com]

Next, we examined whether the distal and proximal CA3 subregions also responded to the novel positional cue exposure. Whereas in the distal CA3 region, no significant change in Arc mRNA expression was detected (Figure 3a,e) (average number of cells analyzed: 11.29 ± 0.32) (one way ANOVA: *p* > .05), we observed a significant increase of Arc mRNA in the proximal CA3 region in animals that explored the novel positional cues (Figure 3b,f) (average number of cells analyzed: 11.67 ± 0.43; Bonferroni test: *p* < .01) compared to control animals.

Overall, there were no detectable changes in Arc mRNA expression in the lower or upper blade of the dentate gyrus (Figure 3c,d,g,h) (one way ANOVA: *p* > .05 vs controls) following positional cue exploration. These data support that the distal CA1 and proximal CA3 regions specifically process “what” information related to positional cues. Interestingly, the dentate gyrus does not respond to this kind of information.

### 3.3 Exploration of large landmarks increases neuronal immediate early gene mRNA expression in the dentate gyrus and CA3 region, but not in the CA1 region

In the past we have observed that novel, spatially arranged, positional cue exposure facilitates the expression of LTD in the CA1 and CA3 regions, but not in the dentate gyrus (Hagena & Manahan‐Vaughan, 2011; Kemp & Manahan‐Vaughan, 2008). By contrast, exposure to novel spatially arranged landmark cues facilitates LTD in the DG, but not CA1 region (Kemp & Manahan‐Vaughan, 2008). Strikingly, the CA3 region also responds to exploration of novel spatially arranged landmark cues with the expression of LTD (Hagena & Manahan‐Vaughan, 2011). Landmark cues can be expected to convery a mixture of “what” and “where” information. Our findings to date, suggest that the hippocampal subfields do not respond in an indiscriminate manner to “what” information, rather the hippocampal subfields may respond to specific aspects or elements of a “what” experience.

**Figure 4:**
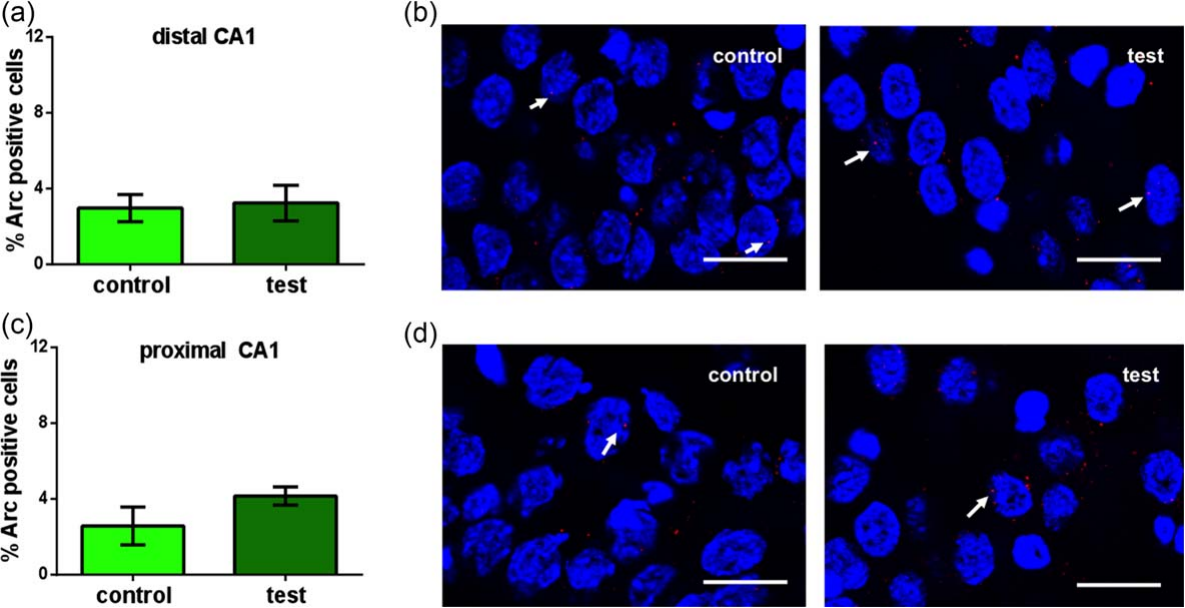
Landmark cue exploration does not trigger immediate early gene mRNA expression the CA1 region. Bar charts show Arc mRNA expression in the CA1 subregions following landmark cues exploration. Novel exploration of spatially arranged landmark cues has no effect on Arc mRNA expression in the distal (a) or proximal CA1 (c) regions. Images show Arc mRNA (red points, indicated by white arrows) expression in the distal (b) and proximal CA1 (d) regions of control animal (control) and animal that explored the landmark cues. Blue: nuclear staining with DAPI. Images were taken using a 63× objective. Scale bar: 10 μm [Color figure can be viewed at http://wileyonlinelibrary.com]

Given the absence of a response of the DG to novel positional cues, we thus explored whether the DG, CA3, or CA1 regions exhibit sensitivity to “what” information that is presented in the form of directional, landmark‐type information. In contrast to the effects of positional cues, no changes in Arc mRNA were detected in the distal (Figure 4A,B) (one way ANOVA: *p* > .05 vs. controls, *n* = 9) or proximal CA1 regions (Figure 4c,d) (one way ANOVA: *p* > .05 vs. controls, *n* = 9) following novel landmark exposure, compared to control levels. (Average number of cells analyzed in distal CA1: 20.78 ± 0.67; in proximal CA1: 20.80 ± 0.76).

**Figure 5:**
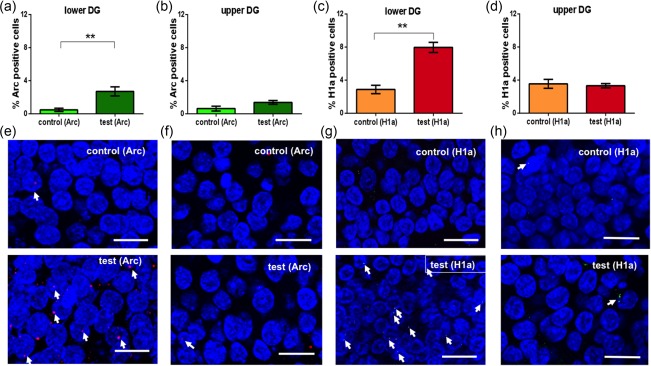
Immediate early gene mRNA expression is increased in the lower blade of the dentate gyrus after landmark cue exploration. The dentate gyrus upper blade is unaffected. Bar charts summarize the immediate early gene mRNA expression in the upper and lower blade of the dentate gyrus. (a) Novel exploration of spatially arranged landmark cues increases Arc mRNA expression in the lower blade of the dentate gyrus but not in the upper blade (b). This latter effect was further verified by scrutinizing Homer1a mRNA expression (c,d). Here, novel landmark exploration triggered increased Homer1a mRNA expression in the lower (c) but not the upper blade (d) of the dentate gyrus. One way ANOVA: *p < .05; **p < .01. (e–h) Images show Arc mRNA (red points, indicated by white arrows) and Homer1a mRNA (green points, indicated by white arrows) expression in the lower blade and upper blade of the dentate gyrus. Blue: nuclear staining with DAPI. Images were taken using 63× objective. Scale bar: 10 μm [Color figure can be viewed at http://wileyonlinelibrary.com]

In contrast, the dentate gyrus exhibited subregion‐specific elevations in Arc mRNA following landmark exposure (*n* = 9). Here, we detected a significant increase in Arc mRNA in the soma of granule cells of the lower blade of the dentate gyrus (Figure 5a,e; one way ANOVA: *F*_1,16_ = 16.16199, *p* < .001 vs. controls, *n* = 9), (average number of cells analyzed: 62.11 ± 2.15), whereas no significant changes occurred in neurons of the upper blade (Figure 5b,f; average number of cells analyzed: 63.97 ± 2.37) (one way ANOVA: *p* > .05 vs. controls). A post‐hoc test also revealed that landmark exposure resulted in significantly higher Arc mRNA levels in the lower compared to the upper blade (Bonferroni, *p* < 0.05).

As the relative expression levels of Arc mRNA were low in the dentate gyrus, we repeated the experiment and examined for changes in Homer1a mRNA expression. Here, we also observed a significant increase in Homer1a mRNA in the soma of granule cells of the lower blade of the dentate gyrus following novel landmark exposure (Figure 5c,g; average number of cells analyzed 76.56 ± 1.64) (one way ANOVA: *F*_1,10_ = 40.7257, *p* < .001 vs. controls, *n* = 6). In the upper blade, no changes were detected (Figure 5d,h; average number of cells analyzed: 82.34 ± 2.76) (one way ANOVA: *p* > .05 vs. controls; *n* = 6). When the percentage of Homer1a mRNA positive cells was compared between the lower and the upper blade of the DG, a significantly greater Homer1a effect was detected in the lower blade (Bonferroni test: *p* < .01) compared to expression levels in the upper blade of the DG.

**Figure 6:**
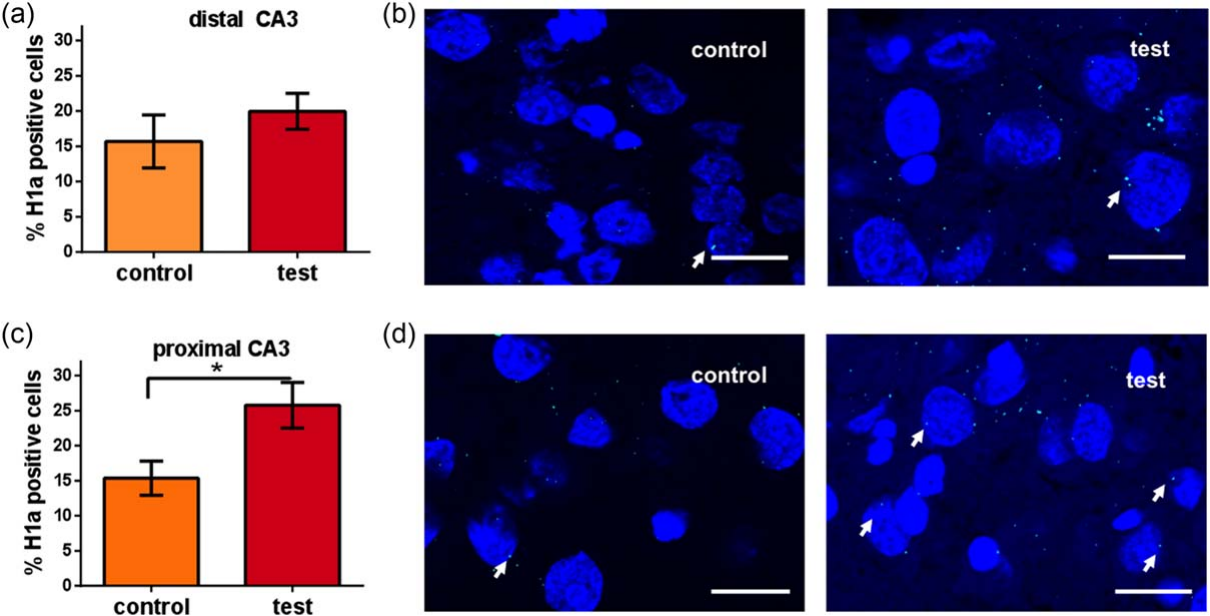
Homer1a mRNA expression is increased in the proximal CA3 region after landmark cue exploration. Bar charts show Homer1a mRNA expression in the CA3 subregions after landmark cues exploration. Interestingly, the proximal CA3 region (c) but not the distal CA3 (a) region responded to the novel landmark exploration. One way ANOVA: *p < .05. Images show Homer1a expression (green points, indicated by white arrows) in the distal (b) and proximal CA3 (d) of a control animal (control) and an animal that was exposed to landmark cues (test). Blue: nuclear staining with DAPI. Images were taken using a 63× objective. Scale bar: 10 μm [Color figure can be viewed at http://wileyonlinelibrary.com]

Additionally, when we quantified Homer1a mRNA expression in CA3 subregions we also observed a significant increase of Homer1a mRNA expression in the proximal CA3 region following novel landmark exposure (Figure 6c,d, average number of cells analyzed: 13.50 ± 0.31) (Bonferroni test: *p* < .05 vs. control, *n* = 6). In contrast, no significant change was detected in the distal CA3 region (Figure 6a,b, average number of cells analyzed: 11.01 ± 0.35, one way ANOVA: *p* > .05 vs. controls; *n* = 6).

Taken together, these data indicate that information that is presented in the form of novel landmark cues specifically targets neurons of the lower blade of the dentate gyrus and the proximal CA3 region. The CA1 region does not respond to this kind of information, at least when it is offered in the form of directional (landmark) cues.

## 4 DISCUSSION

The important role of the medial temporal lobe and, thus, of the parahippocampal formation and the hippocampus in memory function has long been known. But in order to fully understand how information encoding and storage occurs within the hippocampus, it is essential to identify the specific roles of the different hippocampal subregions. Our current results demonstrate that a functional distinction of the hippocampal subregions takes place with regard to the processing of visual information that falls into the “what” category: whereas the CA1 and CA3 regions respond to subtle positional “what” information, the dentate gyrus (and CA3 region) responds to more overt “directional” forms of “what” information that is inextricably embedded in a “where” context. Furthermore, we observed a further specialisation of these subfields with regard to their respective anatomical subcompartments, whereby positional information is processed by the distal and not the proximal CA1 region, and by the proximal but not distal CA3. By contrast, discrete directional information is processed by the lower and not the upper blade of the dentate gyrus and is *also* processed by the proximal CA3 region. These results align very well with the postulates of the parallel map theory of hippocampal information processing, whereby the dentate gyrus processes landmark cue/bearing map aspects of a spatial representation, the CA1 region processes local positional cue/sketch map aspects, and the CA3 region serves as an integrator of both elements of the spatial map. Our results also align with the proposed functional distinction (at the level of anatomical subcompartments) of “what” and “where” visual information processing within the hippocampus, whereby this kind of information is processed by the proximal CA3, distal CA1, and possibly by subcompartments of the dentate gyrus.

For the correct interpretation of our results it was essential to be sure that the rats actually learned in the novel spatial context: we confirmed that exposure to novel directional or positional cues created memories that endured for at least 24 h in our rats. Arc mRNA expression in the hippocampus is related to learning (Bramham et al., 2008; Chawla et al., 2005; Guzowski & Worley, 2001; Guzowski et al., 1999, 2005; Korb & Finkbeiner, 2011; Link et al., 1995; Lyford et al., 1995; Vazdarjanova et al., 2002). Similar evidence has been provided for Homer1a (Vazdarjanova et al., 2002). Here, we observed that whereas the exploration of small (discrete) positional cues resulted in increased Arc mRNA expression in the CA1 and CA3 regions, the exploration of discrete directional cues in the form of landmarks resulted in increased Arc mRNA (and Homer1a) expression in the dentate gyrus and CA3. By “positional” cues, we mean visuospatial cues that can only be detected or processed if the animal is virtually on top of them (spatial microscale), whereas by directional cues we mean cue that can be perceived and processed from afar (spatial macroscale). According to the parallel map theory of hippocampal function (Jacobs, 2003; Jacobs & Schenk, 2003) it is proposed that whereas directional cues enable an animal to find its bearing in an environment, positional cues allow it to create a mental sketch of the feature content if the environment. The parallel map theory also proposes that whereas positional cues are encoded in the CA1 (enabling the generation of a sketch map), and directional cues are encoded in the dentate gyrus, (enabling the generation of a bearing map), the CA3 region serves as an integrator of both elements of the spatial representation. Our results align closely with this hypothesis.

Interestingly, the anatomical segregation of positional cue processing in the CA1 and CA3 regions and directional cue processing in the dentate gyrus and CA3 region, that we observed in this study, is supported by other studies on the interrelationship between subregion‐specific forms of synaptic plasticity and specific elements of a spatial representation: very long lasting forms of LTD are facilitated in the CA1 and CA3 regions when rats explore novel visuospatial positional cues (Hagena & Manahan‐Vaughan, 2011; Kemp & Manahan‐Vaughan, 2004; Manahan‐Vaughan & Braunewell, 1999). In contrast to the facilitation of CA1 LTD by novel positional cues, persistent LTD is enabled in the dentate gyrus, but also in the CA3 region, by novel exploration of *directional* cues (Hagena & Manahan‐Vaughan, 2011; Kemp & Manahan‐Vaughan, 2008). Most strikingly, the subregion and cue‐specific facilitation of LTD in either the CA1, CA3, or dentate gyrus is enabled by exactly the same behavioural paradigms tested in this study (Kemp & Manahan‐Vaughan, 2008).

Our data not only offer empirical support for the parallel map theory, they also offer new insights as to the anatomical subcompartments of the hippocampus that engage in the processing of novel “what” information. The distinction between a “what” stream and a “where” stream in recognition memory was originally proposed for the parahippocampal region (Mishkin et al., 1983). The hypothesis postulates that the perirhinal cortex and the lateral entorhinal cortex principally process information related to an item's features (“what” stream). In contrast, spatial information (“where” stream) is processed principally in the postrhinal cortex and the medial entorhinal cortex (Deshmukh & Knierim, 2011; Furtak, Wei, Agster, & Burwell, 2007; Fyhn, Molden, Witter, Moser, & Moser, 2004; Hargreaves, Rao, Lee, & Knierim, 2005; Kerr, Agster, Furtak, & Burwell, 2007; Young, Otto, Fox, & Eichenbaum, 1997). Both types of information are then integrated in the hippocampus. According to various anatomical and functional data the distal CA1 (close to the subiculum) and the proximal CA3 (close to the dentate gyrus) preferentially process an item's features (“what” stream). In contrast, the proximal CA1 (close to CA2) and the distal CA3 (close to CA2) preferentially process spatial information (“where” stream) (Amaral & Witter, 1989; Burke et al., 2011; Henriksen et al., 2010; Ishizuka et al., 1990; Ito & Schuman, 2012; Sauvage et al., 2013; Witter, 2007; Witter et al., 2000). The present data support the idea that the distal CA1 and proximal CA3 regions preferentially process “what” information. Indeed, the increased Arc mRNA expression induced after exploration of small environmental features was specifically observed in the distal CA1 region, but not in the proximal CA1 region, and conversely was observed in the proximal but not distal CA3. This is also in line with the observation that novel object exposure leads to increased c‐fos expression in the distal CA1 region, whereas novel place exposure leads to increased c‐fos throughout the whole CA1 region (Ito & Schuman, 2012).

With regard to the dentate gyrus, anatomical studies suggest that the lateral entorhinal cortex projects primarily to the upper blade and that the medial entorhinal cortex projects mainly to the lower blade (Tamamaki, 1997; Wyss, 1981). This would suggest that the upper blade preferentially processes “what” information and the lower blade of the dentate gyrus processes “where” information. This assumption is not supported by behavioral studies that examined experience‐dependent Arc mRNA expression in the dentate gyrus: a brief spatial experience (involving novel navigation in an open field environment, which could be interpreted as “where” encoding) induced increased Arc mRNA expression in the *upper* blade, but not the lower blade of the dentate gyrus (Chawla et al., 2005).

However, in the Chawla study, no ostensible landmark (discrete directional) cues were available *within* the environments: the two environments were composed of a square box with high walls, and a rectangular platform, that permitted views of distal cues in the room. An interpretation that could serve to reconcile the results of the Chawla with the abovementioned Tamamaki (1997) and Wyss (1981) studies, is that, as proposed in the parallel map theory (Jacobs & Schenk, 2003), dentate gyrus‐encoded information may take the form of *discrete* directional cues (e.g., visible landmarks), or *distributed* directional cues (Jacobs, 2012; Jacobs & Menzel, 2014), such as odor gradients, or space, the shape of which is polarized (e.g., rectangular). In the Chawla study, the shape of the environment may have had a predominant impact on upper or lower blade encoding. Physiologically this is plausible: hippocampal boundary vector cells (Barry & Burgess, 2014) serve as sentinels of the dimensions of space. In the Chawla study, the change in the environment from square to rectangular in shape, primarily entailed information derived from *distributed* directional cues. In our study, the arena was invariantly square in shape, but the *discrete* directional (landmarks) cues changed their locations. In this case, elevations of both Arc and Homer1a mRNA expression occurred following novel exploration of large landmarks, that specifically took place in the lower blade, and not the upper blade of the dentate gyrus. Thus, a differentiation by the dentate gyrus may occur whereby *distributed* directional cue information is preferentially encoded by the upper blade, and *discrete* directional cue information is encoded by the lower blade. This interpretation would serve to align the results of our and the Chawla studies with the anatomical imputations of Tamamaki (1997) and Wyss (1981) with regard to the role of the upper and lower blades in space encoding. Interestingly, we also observed an increase in IEG expression in the proximal CA3 region following landmark exploration. This suggests, that this kind of information may be perceived as having a strong “what” component (Sauvage et al., 2013; Witter, 2007; Witter et al., 2000), and also adds evidence to the postulate that the CA3 serves as an integrator of information that is processed in a differentiated manner by the dentate gyrus and CA1 regions (Jacobs & Schenk, 2003).

## 5 CONCLUSION

In this study we analyzed the effect of exploration of visual cues that provide positional or directional information, on somatic IEG mRNA expression in the CA1, CA3, and dentate gyrus. Our data provide evidence in support of the parallel map theory and the “two streams” hypothesis: whereas positional cues triggered IEG expression in the CA1 and CA3, but not the dentate gyrus, directional cues trigger IEG expression in the dentate gyrus and CA3, but not in CA1. Both the positional and directional cues contained “what” (item) information, and we observed that the IEG expression triggered in the CA regions was localized to the distal CA1 and proximal CA3, whereas in the dentate gyrus it was localized to the lower blade, and also to proximal CA3. These results indicate that a functional differentiation occurs in the hippocampal subregions with regard to the processing of positional and directional information and that “what” information is processed in a context‐specific manner by the distal CA1 region, proximal CA3 region and the lower blade of the dentate gyrus, whereby the CA3 region is likely to function as an integrator of these different elements of a spatial representation.

## Conflict of interest

None.

## AUTHOR CONTRIBUTIONS

D.M.‐V. devised the concept and experimental strategy of the study. Experiments were conducted by T.‐H.H and V.A. Data analysis and interpretation were conducted by all authors. D.M.‐V. wrote the paper, with contributions from V.A. and T.‐H.H.
